# Supplementary material for: Patterns of Intron Gain and Loss in Fungi
Source: PLoS Biol. 2004 Nov 30;2(12):e422. doi: 10.1371/journal.pbio.0020422 (PMC532390; doi:10.1371/journal.pbio.0020422)
Supplement: Table S1 — Also available at http://genes.mit.edu/NielsenEtAl/. (4.3 MB ZIP). [file pbio.0020422.st001.zip › NielsenEtAl/html/1206.html]

AN1189.1.NCU04736.1.MG02487.1.FG09515.1


```
 CLUSTAL W (1.82) Multiple Sequence Alignments - Introns Inserted


Sequence 1: MG02487.1	1447 aa
Sequence 2: FG09515.1	1340 aa
Sequence 3: NCU04736.1	1385 aa
Sequence 4: AN1189.1	1432 aa
Alignment Length: 1496 aa
Number Identitical Residues: 591 aa
Alignment Score (without introns) 29079


MG02487.1 	---------MADNKDDGATPPRRQRA2PTITIDTTAVSASTDAGAPADRPSQANGQTSSP
NCU04736.1	--------------------------~---------------------------------
FG09515.1 	---------MADHDDN---PLMRRRA2PTITIDTTAVNPNTSTDPPQE---------SHP
AN1189.1  	MSEPRQSSENNDSRQPASTPLRRERA~PTITIDTSAVVSSEPPPQIEAPSQSSQSRSAYN
          	 :.. .::.  .  . .::.     : .: : .:::  ..  .      ...:.. ::  

MG02487.1 	ISPTDDGNSPTTIVEPLSQSWSRGQPSNGGHSASSSISGRPEFHPTTSFESNASRPTSPH
NCU04736.1	---MDPIASHWTPSHQHSNSLGQ----------------RSEMRQSTSFDSRDSRPTSPH
FG09515.1 	SHLQDDAVSPTSTADPAATAGMSQRPS------------RPEVNTSLSFENRDSRPTSPH
AN1189.1  	ADHTDTSALLNSSTVSPSDTRSAH---------------SVRSYASSEGREHDSRPTSPS
          	    *      :     : :                     .   : .  .. ****** 

MG02487.1 	NVSSPVASRGQGHTQGFLAVP-HNHRSRQNSVDSDDGSRSMAS-QGETIAVSPSYTTEKA
NCU04736.1	NVSSPVTYRPMD-PQNFLSVPGAAGRSRQNSVNSNDETRSIASSQGETVIGGSSHQGDKI
FG09515.1 	NVSSPVLSRSGDKGVEFLSVP-INHRSRQNSVDSDDMSRSVSS-QGDTTVVASNSTQHET
AN1189.1  	PRTNTFSPGAKMGDSNYLSVP--GTRSRGNSLESEDSSHTIGA---ESRSIGSHGSPASS
          	  :...          :*:**    *** **::*:* ::::.:   ::   ..     . 

MG02487.1 	PK--STDPMTNDKIMNDPDALKPDVGKEDTFVVEDNPFAFSPGQLSKMFNPKSLSAFYKL
NCU04736.1	GRPLSSNTSDNAQIMNDPDALKPDQGREADFEVVDNPFAYTPGQLNKMFNPKSLSAFWKL
FG09515.1 	LK--GSE-YDHNKIINDETALKPDTGTEQDFEVENNPFAFAPGQLNKMFNPKSLSAFYKL
AN1189.1  	AK---------VTIENYEEALLPDPGREAEFEVENNRFAFSPGQLNKLLNPKSLSAFYAL
          	 :           * *   ** ** * *  * * :* **::****.*::********: *

MG02487.1 	GGLSGLEKGLRTDRKAGLSVDELGLEGKVTFEEVTG-QKSEKETNTGAGKPQANRARSGS
NCU04736.1	GGLAGLEKGLRTDRKAGLSMDEVEVDGRVTFDEATA-RPSGNPPAQPKAATETVLAKTDS
FG09515.1 	GGIDGIEKGLRSDRKAGLSIEEKSLGGQVSFEDATS-KKQ---------TPHNDVSNTQP
AN1189.1  	GGLAGLAKGLRTDPRSGLSLDETELDGSVSFEDATAPSNNQPLPKPAAEAPPAEPSRADT
          	**: *: ****:* ::***::*  : * *:*::.*..  .   .      .    :.: .

MG02487.1 	------------EDNFVDRKRVYKDNRLPERTGKSLLQLMWITFNDKILLLLSGAAAISL
NCU04736.1	HAAADHHKKHHGDDHYTSRKRVFSDNRLPEKKGKSLLELMWITYNDKVLILLSIAAVVSL
FG09515.1 	------------GDSFADRLRIYKDNRLPEKKGKSLLQLMWITYNDKVLILLSIAAVVSL
AN1189.1  	TPHK------QDENAYSDRKRVFGANKLPEKKTKSILELAWLAYNDKVLILLTVAAIISL
          	 .         . : : .* *::  *:***:. **:*:* *:::***:*:**: ** :**

MG02487.1 	AVGLYEAFSPDHDPSKQK0VEWIEGVAIIVAILIVVLVGSLNDWQKERQFAKLNKKKTDR
NCU04736.1	AIGLYQTFGQAHKPGEAK~VEWVEGVAIIVAIVIVVMVGSLNDYQKERQFAKLNKKKQDR
FG09515.1 	AVGLYQTFGGEHKDGEPK~VEWVEGVAIIVAIAIVVIVGSLNDYQKERQFTKLNKKKQDR
AN1189.1  	ALGIYQSVT--AVPGEPR~VQWVEGVAIIVAILIVVVVGAANDWQKERQFVKLNKKKEDR
          	*:*:*::.      .: : *:*:********* ***:**: **:******.****** **

MG02487.1 	PVKVIRSGKAQEISVHNLLVGDVVHLETGDLIPVDGVLIEGHNIKCDESQATGESDLIKK
NCU04736.1	LVKAIRSGKTVEISVFDVLVGDVLHLEPGDMIPVDGILIEGYNVKCDESQATGESDIIRK
FG09515.1 	LVKVIRSGKTIELSVFDILAGDVVHLEPGDLVPVDGILIEGFNVKCDESQATGESDIIRK
AN1189.1  	LVKVIRSGKMIEISIHDILVGDVMHLEPGDLVPVDGIYIGGHNVKCDESSATGESDVLRK
          	 **.*****  *:*:.::*.***:***.**::****: * *.*:*****.******:::*

MG02487.1 	RNADEVYAAIENNGDLKKMDPFIQSGARVMEGVGTFMVTATGVNSSYGQTLMSLQDDPEI
NCU04736.1	RPADEVYAAIENNENVKKMDPFIQSGARVMEGMGTYLVTSTGIYSSYGRTLMALDEDPEM
FG09515.1 	QAAEVVYNAIENRDNLKKMDPFIQSGARIMEGVGTYMATSVGVYSSYGKTLMSLNEDPEM
AN1189.1  	TPAQDVYGAIERHENLAKMDPFIVSGAKVSEGVGTFLVTAVGVHSTYGKTMMSLQDEGQT
          	  *: ** ***.. :: ****** ***:: **:**::.*:.*: *:**:*:*:*::: : 

MG02487.1 	TPLQQKLNVIADGIAKIGGAAALLLFLVLFIKFLVGLPKQPPEITPAQKGQQFIRIFIVV
NCU04736.1	TPLQSKLNVIAEYIAKLGGAAGLLLFIVLFIIFLVKLPK--SQHTPAEKGQQFLNIFIVV
FG09515.1 	TPLQAKLNVIATYIAKLGSAAGLLLFIVLFIKFLVGLPKMGPGVSPAQKGQQFLNIFIVV
AN1189.1  	TPLQTKLNVLAEYIAKLGLAAGLLLFVVLFIKFLAQLKS---LGNADEKGQAFLQIFIVA
          	**** ****:*  ***:* **.****:**** **. * .     .. :*** *:.****.

MG02487.1 	VTIIVVAVPEGLPLAVTLALSYATKKMVKQNNLVRQLKACEVMGNATTICSDKTGTLTQN
NCU04736.1	VTIIVVAVPEGLPLAVTLALAFATTRMLRDNNLVRHLKACEVMGNATTICSDKTGTLTQN
FG09515.1 	VTIIVVAVPEGLPLAVTLALAFATTRMLRDANLVRHLKACEVMGNASTICSDKTGTLTQN
AN1189.1  	VTVIVVAVPEGLPLAVTLALAFATTRMLKDNNLVRLLRACETMGNATTICSDKTGTLTEN
          	**:*****************::**.:*::: **** *:***.****:***********:*

MG02487.1 	KMKVVEGTVGTTHRFSADDFSTGDSE-NPSSPEVKP--LSPTEFTGLLSEPVKEVLLRSI
NCU04736.1	KMQIVAGTVGTTHRFGGVG-SAGGLN-PETPDSPTEADVTAKEVVASLDASVKELLLKSI
FG09515.1 	KMQVVSGTIGTSLRFGGS--QRGDDS-NASTPVDTSGDISIGEFAKMLSKPVKDILLKSI
AN1189.1  	KMTAVAATLGTGTRFGGRSQAASPTNRNGDRPADSGNELSPSEFASSLSKPAKELLLDSI
          	**  * .*:**  **.. .   .  .        .  .::  *..  *. ..*::** **

MG02487.1 	VLNSTAFEGEVDGEQSFVGSKTETALLLFAREHLAMGSVSEQRENSKTLQLIPFDSGRKC
NCU04736.1	SLNSTAFEGEIDGVKSFVGSKTETALLEFAKEHLAMGPIAEERANAKILHLIPFDSGRKC
FG09515.1 	ALNSTAFEGEVDGEKTFIGSKTETALLILAKSHLGMGPVSEERENAKVLQLIPFDSGRKC
AN1189.1  	VLNSTAFEGEQEGTMTFIGSKTETALLGFARTYLGLGSLSEARDNASIVQMVPFDSGRKC
          	 ********* :*  :*:********* :*: :*.:*.::* * *:. ::::********

MG02487.1 	MGIVAELPKGG-ARLYVKGASEILLEKCTQIIRDPSKDATTATLTDENRTGLNSLIENYA
NCU04736.1	MGVVVALDNGK-ARLYVKGASEIMLEKCTQILRDPSNGITAGPLTQENRETVLKLIETYA
FG09515.1 	MGIICQGPNGS-ARLYIKGASEIILSKCTQVFRDPANDDSLAPMSDDNTETVKQLIESYA
AN1189.1  	MAVVIKLDNGKKYRMLVKGASEVLLAKSTRIVRNPTQNLEEGPLDDKDRSKLDETINKYA
          	*.::    :* . *: :*****::* *.*::.*:*::.   ..: :.:   : . *:.**

MG02487.1 	KKSLRTIGICYRDFDRWPPARARRNDGENDEVKFEDIFKQMTLLGVVGIKDPLRDGVREA
NCU04736.1	RNSLRTIGIIYRDFAHWPPAKARRTGEDKEEIVFEDICSQMTFVGMVGIKDPLRPGVPEA
FG09515.1 	RRSLRTIGICYKDFPSWPPKNVSHIDG-GKEVVFEDIFMDMAFIGVVGIQDPLREGVPEA
AN1189.1  	TQSLRTIGLVYRDFTEWPPRGAPTQEEDRSLAAFDSIFKDMVMFGVFGIQDPLRAGVTES
          	 .******: *:**  ***  .     . .   *:.*  :*.:.*:.**:**** ** *:

MG02487.1 	VKDCQRAGVVVRMVTGDNIMTAEAIARDCGILQPDSIIMEGPKFRNLSKREQEDVVPRLH
NCU04736.1	VQLCQKAGVVVRMVTGDNKITAEAIAKDCGILQPNSLVMEGPEFRNLSKAKQEEIIPRLH
FG09515.1 	VKLCQKAGVTVRMVTGDNKITAEAIAKECGIIQPNSIVMEGPEFRNLSKLQQEEIIPRLH
AN1189.1  	VQQCQRAGVFVRMVTGDNIVTAKAIARECGIFTPGGVAIEGPKFRKLSSRQMTQIIPRLQ
          	*: **:*** ******** :**:***::***: *..: :***:**:**. :  :::***:

MG02487.1 	VLARSSPEDKRVMVKRLKDKGHIVAVTGDGTNDAPALKMADVGFSMGVSGTEVAKEASAI
NCU04736.1	VLARSSPEDKRILVKRLKDMGEIVAVTGDGTNDAPALKMADVGFSMGIAGTEVAKEASAI
FG09515.1 	VLARSSPEDKRILVKRLKDKNETVAVTGDGTNDAPALKMADVGFSMGIAGTEVAKEASAI
AN1189.1  	VLARSSPDDKKILVSQLKKLGETVAVTGDGTNDAQALKTADVGFSMGITGTEVAKEASDI
          	*******:**:::*.:**. .. *********** *** ********::********* *

MG02487.1 	ILMDDNFTSIVVALRWGRAVNDAVKRFLQ~FQLTVNVTAVLLTFVSAVSNDREESVLTAT
NCU04736.1	ILMDDNFNSIVKALKWGRAVNDAVKRFLQ~FQLTVNVTAVILTFISAVSNKEQDSVLTAV
FG09515.1 	ILMDDNFNSIVKALKWGRAVNDAVKRFLQ~FQLTVNITAVILTFVTAVSNDEESSVLTAV
AN1189.1  	ILMDDNFASIVKAMAWGRTVNDAVKKFLQ0FQITVNITAVLLTFISAVASGDEESVLTAV
          	******* *** *: ***:******:*** **:***:***:***::**:.  :.*****.

MG02487.1 	QLLWVNLIMDTLAALALA~T0DPPHPTVLDRKPEPRGSPIISVTMWKMILGQALYQLGIT
NCU04736.1	QLLWVNLIMDTLAALALA~T~DPPADSVLDRKPERRGSGIISTTMWKMIIGQAIYQLAIT
FG09515.1 	QLLWVNLIMDTLAALALA~T~DPPHDSVLDRKPEPKGSSIISVTMWKMILGQSVYQLVIT
AN1189.1  	QLLWVNLIMDTFAALALA1T~DPPSPYVLNRRPEPKSAPLINLTMWKMMIGQSIYQLVVT
          	***********:****** * ***   **:*:** :.: :*. *****::**::*** :*

MG02487.1 	YLLYFGRQRVLPAYDQDVQDA----QIATLVFNTFVWMQIFNQWN2NRRLDNRFNILEGI
NCU04736.1	LLIYFGKQGVLPNYDDNVTDD----QIQTLVFNTFVWMQIFNQWN2NRRLDNNFNIFEGL
FG09515.1 	FLLYYGGKDLVPTKN-TPSDD----EIKTLVFNTFVWMQIFNQWN2NRRLDNKFNIFEGL
AN1189.1  	LVLNFSGRSILKSIIDFSGDANANNVLTTVVFNTFVWMQIFNQWN2SRRLDNGLNIFDGL
          	 :: :. : ::    .   * .:.. : *:*************** .***** :**::*:

MG02487.1 	TKNPIFIAISLAMCGAQALITNYGSFAFNIAEEGQTPAMWGYAIFLGFLSIPFGMIIRLI
NCU04736.1	TKNLFFLGISAIMMGGQVLIVFVGGQAFSIAKEKQTGAMWAYALILGFISIPVGMIIRLI
FG09515.1 	TKNWFFISISAIMCGGQVLIIFVGGAAFQIAKEKQSGALWAIALVLGFLSIPVGILIRLI
AN1189.1  	FRNRWFIGIQFIIVGGQILIIFVGGHAFSVTR--LTGAQWAVCLILGVISIPVGVIIRLI
          	 :*  *:.*.  : *.* **   *. **.::.   : * *. .:.**.:***.*::****

MG02487.1 	PDAFVASLVPESLRRRARSKVPGVTVSDDEERFQAYPEPFSEVRDELAFIKRVKGGRLNN
NCU04736.1	PDSLFERMVPEYIKRRAN-KTPDLTVSDDE-RFEYYPPAFAEVRDELAFLKQFKGGRINN
FG09515.1 	PDHFLLVLVPEFLKQRAN-KVPGLTVSDEE--MSMYPEPLADVRDELNFMRRMKGGRLNN
AN1189.1  	PDEFIRKLIPTFHRKKGP----ELIVSDEDRRFEWNP-ALEEIRDQLKFFKSLRGGRLRK
          	** :.  ::*   :::.      : ***::  :.  * .: ::**:* *:: .:***:.:

MG02487.1 	LKYSLKNPREAFMPRYKSPSGSRSHSRSHSRSDSIPMPTTPTRADSISSF----AATPDS
NCU04736.1	LKFAMKHPRETFMPK-----RSASHSRSNSKSNSINVPMTPIRKDSTGGAPSIAPPTPDS
FG09515.1 	LKFAIQHPKEAVEM-------VRSRSPSRSRSGSVNAPQTPTQENPFEGS----PIGTPN
AN1189.1  	VAHKLQHPQELLPR-----------SRSGSRSRENSAPGTPVGDSSEGTPP--FSASPDS
          	: . :::*:* .             * * *:* .   * **   ..    .   .  . .

MG02487.1 	RRRSKSSRSRSNSALGAATVMAGIVAGGVGATWSP--IDRDRDFGVFPARQPSP-LNKRQ
NCU04736.1	RRRSRSTRSRSNSALGASMVMTGIIAGSVAAGWAPSPVERRPDSDFGLFPPKSS-PHAES
FG09515.1 	SVRTRSTRSRSNSALGAPTVMAGIVAAGVAAGWQP--KMR-------PADDGDN-TNAQK
AN1189.1  	RSR-RRTRSRSNSAFGPAAAMAGVVAGSIAG-WSP--IERPGEGEAFKFDFNSNGRRSGQ
          	  * : :*******:*.. .*:*::*..:.. * *    *  .         . . .  .

MG02487.1 	DSDPMDGAGASTSNVSGMSVPEESNEEDKPGPTVPKLGVPKPPPGRKSHESSKE------
NCU04736.1	SQSEVQPSQRSLTPSINEEQETPDIVPSIVEQTVPILSVPKPPGQKSA------------
FG09515.1 	------------------------------------------------------------
AN1189.1  	PGIEVHPDTAPDDNIIGDYQATSTTPPSQNPDLIPFFEHAPPAREPSHRSRRSRSRSSQS
          	          .     .          .      .    . ..   .  .  . : ::.:

MG02487.1 	--
NCU04736.1	--
FG09515.1 	--
AN1189.1  	QS
          	.:
```
